# Companion Dogs and Cats as Key Reservoirs of Antimicrobial Resistance: Evidence and One Health Implications

**DOI:** 10.3390/antibiotics15050515

**Published:** 2026-05-19

**Authors:** Balamuralikrishnan Balasubramanian, Sureshkumar Shanmugam, In Ho Kim

**Affiliations:** 1 Department of Food Science and Biotechnology, College of Life Science, Sejong University, Seoul 05006, Republic of Korea; 2 Department of Animal Resource and Science, Dankook University, Cheonan-si 31116, Republic of Korea

**Keywords:** antimicrobial, resistance, companion animals, antibiotics and infections

## Abstract

Antimicrobial resistance (AMR) in companion animals is an escalating concern at the interface of veterinary medicine and public health. Dogs and cats, the most commonly treated companion species, are frequently prescribed antimicrobials for dermatological, otic, urinary, and respiratory infections—often involving drug classes that are critically important in human medicine. This overlap underscores the need for judicious use and integrated stewardship within a One Health framework. This narrative review synthesizes current evidence on AMR in companion animals and its implications for One Health. Studies were included if they reported AMR in dogs and cats and addressed zoonotic aspects. *Staphylococcus pseudintermedius*, *S. aureus*, *Escherichia coli*, *Pseudomonas aeruginosa*, and *Enterococcus* sp. are examples of clinically significant organisms that are becoming more resistant to several antibiotic classes, which can result in treatment failures and extended illness. Horizontal gene transfer facilitates the spread of resistance determinants across bacterial populations. Improved surveillance systems, prudent antibiotic use, regular culture and susceptibility testing, and enhanced antimicrobial stewardship in veterinary practice are just a few of the many strategies needed to address AMR in companion animals. The integration of companion animals into AMR surveillance, stewardship programs, and infection control strategies is essential. Coordinated One Health interventions are urgently required to mitigate the spread of AMR.

## 1. Introduction

The concept of pet humanization has become a trend in which companion animals obtain more concern, care, and attention, and is another outcome of the rising bond between people and their pets [1]. According to a recent survey, 97% of pet owners consider their animals to be members of the family, while others see them as an extension of themselves [2]. Concerns about the health and welfare of pets have grown dramatically as a result of this trend, which has led to higher expenditure on veterinary care and diagnostics [3]. One of the most prevalent conditions in companion animal medicine is skin and ear infectious diseases, which are clinically manifested as multisystemic dysfunction [4]. The etiology is typically identified with a thorough physical examination and simple point-of-care testing. Infectious etiologies account for a significant percentage of dermatology consultations and ear disease presentations, frequently as a result of underlying predisposing conditions [5]. Ear infections chiefly include otitis externa and related ear infections; however, studies have shown differences in prevalence depending on geographical region and across the breed of dogs. For instance, an epidemiologic study of dogs in Northwest China reported that 1.0% of visits were for ear disease, amongst them 84.6% corresponded to otitis externa. Moreover, for breed predisposition, it was evident that Toy Poodles, Cocker Spaniels, and Golden Retrievers reported for 18.5%, 10.4%, and 9.5% of the cases, respectively [6]. A more recent review reported that the typical prevalence estimated for canine otitis externa range from 4.6% to 7.3% in general practice; however, a few other studies have reported up to 10–20% of dogs affected [7]. Similarly, another report from India accounted that 14.13% of screened dogs (92/651) had an infection of otitis externa [8].

In a 2024 systematic review and meta-analysis of Korean dogs with pyoderma and otitis externa, the authors reported that a pooled bacterial pathogen pervasiveness of 99.95% was observed in infected dogs, and clinically confirmed the diagnosis that pyoderma or otitis externa had clinical pre-inclination to bacterial pathogens [9]. Pyoderma is the most prevalent bacterial skin infection in dogs, and commonly occurs as secondary to underlying conditions such as allergic disorders or ectoparasitic infestations. In veterinary practice, it represents a leading indication for antimicrobial prescriptions. [10]. Although usually not fatal, if the underlying cause is not addressed, the risk of recurring infections and antimicrobial resistance is increased and alarming [11]. While the detailed prevalence of skin infections in dogs is less firmly quantified, a recent review of bacterial skin infections reported that 80.6% yielded isolates had *S. pseudintermedius*, which was the most common pathogen, accounting for about 37.5% of total skin infections in dogs [12]. Pyoderma exhibits marked variation by age, sex, and geography, with the greatest burden observed in resource-limited settings. Accordingly, priority should be given to policy development and the implementation of targeted, risk-stratified prevention and treatment strategies. Such approaches are essential to effectively reduce disease burden and to address the global impact of pyoderma [13].

Another significant skin infection, which has been reported globally, is atopic dermatitis (AD), which is a prevalent inflammatory and itchy allergic skin condition that affects dogs [14]. The immunologically complex and multidimensional pathophysiology of AD may include immune dysregulation, microbiome alterations, genetic variables, allergy sensitization, and epidermal barrier failure. The goals of the multimodal treatment for canine atopic dermatitis (CAD) include nutritional management, skin barrier repair, and the control of pruritus, related inflammation, and infections [15]. With an emphasis on the disease’s subsequent care in the canine population, information on the prevalence, effects, and intricate immunological interactions of AD should be addressed with a multimodal approach [16]. In a review report of CAD, which often predisposes to skin infections, the authors reported that the prevalence was estimated to be within the range of 3–15% [17]. For a number of reasons, atopic dermatitis is of high clinical significance. It can be difficult to treat, particularly in resistant or recurrent cases; it often recurs and is frequently secondary to other underlying conditions. Moreover, it causes morbidity, itching, pain, discomfort, and hearing impairment, and it may contribute to antimicrobial resistance [18].

The objectives of animal welfare, clinical success, and antimicrobial-stewardship are contingent on the proper use of antibiotics in veterinary dermatology and otology because the skin and ear canals of companion animals are frequently the site of bacterial and fungal infections [19]. The most common causes of superficial pyoderma and otitis externa are *Staphylococcus* sp., *Pseudomonas* sp., and the yeast *Malassezia*. These microorganisms vary in susceptibility and have increasingly been demonstrating multidrug resistance. They are often identified as secondary to underlying diseases, such as allergy, endocrinopathy, and the presence of foreign bodies. Prompt, focused therapy lessens discomfort, halts long-term alterations, like tympanic injury and canal stenosis, and reduces the proliferation of resistant strains [20]. Across the studies, this review has identified the following research gaps in veterinary otology and dermatology despite the growing body of literature. First, surveillance has been fragmented. For instance, regional studies have reported rising antimicrobial resistance in otitis [21] and pyoderma; however, there is no global, standardized dataset to guide empirical therapy and stewardship [22]. Second, the clinical impact of biofilms and persistent *Pseudomonas* infections in chronic otitis needs translational research using in vivo models to understand the outcomes of randomized trials of anti-biofilm strategies. Third, diagnostic gaps persist; although the monotonous use of cytology is practiced and widespread, it lacks susceptibility testing and rapid and accurate reporting standards, which are poorly defined and impact on the outcomes [23]. Fourth, longitudinal, multiomic studies that could identify preventive targets are still lacking in their exploration of the role of the microbiome and host–microbe interactions, including allergic illness modification of the ear/skin environment [24]. This review is structured by framing antimicrobial resistance (AMR) within the global context, then by emphasizing its veterinary relevance in high-use areas, such as dermatology and otology. Companion dogs and cats are defined as reservoirs and transmission interfaces, supported by shared resistant pathogens and horizontal gene transfer. With a focus on antimicrobial resistance trends, diagnostic limitations, microbiome interactions, and therapeutic gaps, this review aims to critically evaluate antimicrobial resistance in canine and feline skin and ear infections, to identify emerging resistance trends and knowledge gaps, and to propose evidence-based strategies to improve clinical outcomes and antimicrobial stewardship within a One Health framework.

## 2. Methodology

A structured literature search was conducted using PubMed, Scopus, and Web of Science for studies published between 2006 and 2025. Search terms included “antimicrobial resistance”, “companion animals”, “dogs”, “cats”, “pets”, “zoonotic transmission”, and “One Health”, combined using Boolean operators.

Studies were included if they reported AMR in companion animals (primarily dogs and cats) and addressed public health or zoonotic aspects. Relevant data were extracted and synthesized qualitatively, focusing on resistance patterns, key bacterial species, and transmission pathways between animals, humans, and the environment. The findings are organized thematically to highlight epidemiology, resistance determinants, transmission, and One Health implications.

## 3. Etiology and Microbial Spectrum

### 3.1. Bacterial Pathogens Associated with Skin Infections

**Figure 1:**
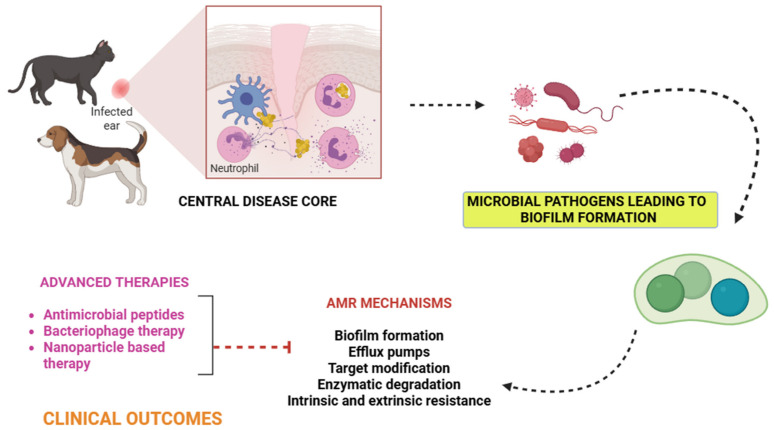
Mechanisms of antimicrobial resistance in skin and ear infections of dogs and cats.

In veterinary medicine, one of the most common reasons for recommending antibiotics is skin and soft tissue diseases, especially pyoderma. Coagulase-positive staphylococci are the major bacterial pathogens responsible for these diseases [25]. Table 1 presents the microbial etiology and AMR Profiles of the ear and skin infections in companion animals. Over 90% of canine pyoderma cases are caused by *Staphylococcus pseudintermedius*, up to 10% of cases are caused by the next most prevalent bacteria, *S. aureus* and *S. coagulans* [26]. By contrast, *S. aureus* is a more common causative organism of pyoderma in cats than *S. pseudintermedius*, despite the fact that there is less information on these cases. It is still debatable how often the comparable bacterial illnesses are in other companion animals, such as rabbits [27]. In a four-year study conducted by the University Veterinary Teaching Hospital of Naples for specific regular bacteriological investigations in companion animals, pyoderma was exposed to be widespread in cats, while otitis externa was the most common cutaneous infection observed in dogs. However, gram-positive strains showed a higher incidence than gram-negative bacteria. *S. pseudintermedius* (65%) and *Pseudomonas aeruginosa* (36%) were frequently reported in dogs; coagulase-negative *Staphylococci* (47%) in cats. The phenotypic profiles of antibiotic resistance reported that these bacterial strains exhibited strong resistance to the majority of antibiotics, such as amoxicillin–clavulanate, penicillin, clindamycin, and trimethoprim–sulfamethoxazole [28]. A study from the Valencia region assessed AMR and multidrug resistance (MDR) in commensal and pathogenic *Staphylococcus* sp. isolated from dogs and cats. Figure 1 shows the mechanisms of AMR in companion animals.

Antimicrobial susceptibility testing of 271 veterinary samples against 20 antibiotics, including last-resort agents, yielded 187 *Staphylococcus* isolates. *Staphylococcus pseudintermedius* predominated in dogs (~60%), while *Staphylococcus felis* was most common in cats (~50%). Resistance was detected to all tested antimicrobials, including those critical for human medicine, with MDR observed in over 70% of canine and 30% of feline isolates. These findings underscore companion animals as important reservoirs of AMR, and highlight the necessity of sustained surveillance within a One Health framework. Development of AMR and MDR in bacteria to commonly used antibiotics is a major concern in both human and veterinary medicine.

Companion animals play a substantial role in the epidemiology of AMR due to their expanding population and capacity to spread AMR species, like methicillin-resistant *Staphylococcus* strains, which are of public health concern [36]. Marco-Fuertes et al. investigated the frequent occurrence of AMR and MDR in commensal and pathogenic *Staphylococcus* sp. in dogs and cats in the Valencia region. The study involved data from 271 samples collected from various veterinary clinics. About 187 samples accounted for the *Staphylococcus* sp. collected from asymptomatic and skin-diseased dogs and cats. Amongst them, *S. pseudintermedius* and *S. felis* demonstrated common incidence of 60% and 50% in dogs and cats, respectively. However, AMR was found for every antibiotic examined in the isolate in the overall analysis, including those that are essential to human treatment [37]. Therefore, the study implied the trends in AMR and MDR among companion animals. Adopting a One Health strategy is crucial because these animals can contribute to the spread of AMR and resistance genes in humans, in other animals, and in their mutual environment. Superficial pyoderma is a common complication for a range of feline and canine skin diseases. A retrospective study [38] evaluated bacterial and fungal skin culture results from superficial pyoderma cases in cats (*n* = 28) and dogs (*n* = 35), focusing on pathogen distribution, antimicrobial susceptibility patterns, and associated clinical and demographic data. *Staphylococcus* sp. and *Trichophyton* sp. were the most frequently isolated microorganisms in both species, with antimicrobial resistance detected across the samples. The findings emphasize the critical role of skin culture and susceptibility testing in guiding effective therapy, optimizing treatment protocols, and mitigating the rising burden of antimicrobial resistance, thereby supporting both animal and public health. A similar study investigated the skin conditions in the superficial pyoderma of infected cats and dogs. The study retrospectively assessed the bacterial and fungal skin culture samples in the above cases and detailed the respective pathogens and their antimicrobial susceptibility resistance, along with clinical symptoms and inclusive patient demographics.

**Figure 2:**
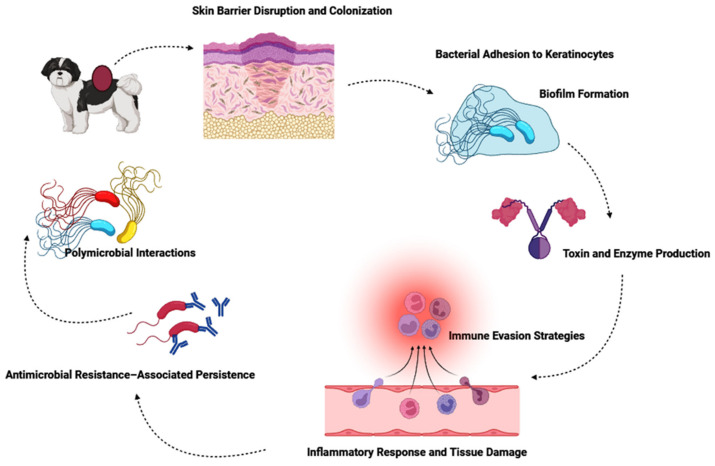
Schematic representation of the major mechanisms involved in the microbial skin and ear infections of companion animals.

The most prevalent cultured bacteria samples were *Staphylococcus* sp. and *Trichophyton* sp., which exhibited antimicrobial resistance to almost all the antibiotics [38]. Hypersensitivities, atopic syndromes, ectoparasites, endocrinopathies, dermatophytosis, and aberrant cornification are the primary disorders linked to pyoderma. According to a recent study, one of the aforesaid underlying diseases was present in 68% of dogs with recurring pyoderma, 59% of allergies being the most frequent. This condition is due to factors like thin and compact nature of canine stratum corneum, with minimal lipid follicular plug and intercellular lipids with low pH which increased the susceptibility to pyoderma. These traits might make it favorable for *staphylococci* to survive and adhere to dog skin [39]. Another type of skin infection observed in companion animals is impetigo, which mainly occurs in newborn puppies and young dogs and is characterized by pustular skin illness; it is also reported in older dogs due to predisposed conditions. The causative organism is *S. pseudintermedius* is an opportunistic commensal [40], and is the primary cause of this non-follicular, subcorneal pustular dermatitis, which is one of the mildest types of canine pyoderma. Although impetigo is usually self-limiting, nonpainful, and nonpruritic, it still needs to be properly diagnosed and treated to avoid developing more serious pyoderma or recurrent infections [41]. Topical antimicrobials are the mainstay of therapy, and treatment is often successful. Preventing recurrence requires addressing the predisposing circumstances. However, impetigo is a benign illness, it is important to understand antimicrobial stewardship, particularly in light of the increasing prevalence of multiple drug resistance. Effective dermatological practice still benefits from ongoing research on resistance patterns and non-antibiotic treatments. However, resistance arises through enzymatic inactivation, target modification, efflux pumps, biofilm formation, and horizontal gene transfer, while polymicrobial interactions are compounded by host-related and therapeutic factors. Figure 2 shows a schematic representation of the major mechanisms involved in the microbial skin and ear infections of companion animals.

### 3.2. Bacterial Pathogens in Ear Infections (Otitis Externa/Media) in Companion Animals

Bacterial infection plays a significant role in both otitis externa, an inflammation of the external ear canal, and otitis media, the involvement of the middle ear, in companion animals, particularly in dogs, and less frequently in cats. This is reported to be the most common complaint in small animal practice [42]. The microbiological diversity, resistance profiles, and clinical etiquette differ by host species, chronicity, clinical history, and other predisposing factors. The current literature has revealed a persistent core of bacterial pathogens, with *Pseudomonas aeruginosa* and *Staphylococcus pseudintermedius* as the core dominating causative organisms; however, a larger diversity of Enterobacteriaceae, *Proteus*, *Klebsiella*, *Streptococci*, and anaerobes are frequently investigated [43]. Table 2 lists the bacterial pathogens associated with skin infections in dogs and cats.

Although otitis media, an inflammatory middle ear infection, is more common in dogs than previously thought, it is commonly disregarded in veterinary treatment. About 16% of acute otitis externa cases and 50–80% of chronic cases result in secondary otitis media [50]. Its significant incidence in chronic otitis externa may cause veterinarians to reevaluate their diagnostic approach, especially when a dog has a history of frequent ear infections. Otitis media should also be suspected in individuals presenting head-related neurological symptoms, such as vestibular dysfunction, Horner’s syndrome, or facial nerve abnormalities. The most frequently isolated microbial species from canine otitis samples across various geographical regions and surveillance investigations is *S. pseudintermedius*, a typical commensal of canine skin and ear canals [51]. It can be a pragmatic and secondary invader in damaged or inflamed tissue, and is associated with both acute and chronic otitis. Methicillin-resistant *S. pseudintermedius* (MRSP) strains are often multidrug resistant and complicate empirical therapy, therefore their emergence and spread have obvious therapeutic implications. MRSP has been identified as a veterinary antimicrobial-resistance priority by regulatory and risk-assessment organizations [52]. Saengchoowong et al. [53] evaluated the bacterial populations in the external ear canals of healthy dogs and dogs with otitis externa (OE). The study involved bacterial samples isolated from four groups of dogs viz., healthy erect-ear dogs, erect-ear dogs with OE, healthy pendulous-ear dogs, and pendulous-ear dogs with OE. The V3/V4 region of 16S rDNA was amplified, sequenced, and analyzed for rarefaction, bacterial taxonomy, relative abundance, richness, and diversity. The results demonstrated that healthy dogs showed greater bacterial richness and variety than OE-affected pups. Consistent with earlier culture-based findings, *Corynebacterium*, *Pseudomonas*, *Staphylococcus*, and *Proteus* sp. were common in OE. High-throughput sequencing also discovered possible new diseases, including *Tissierella*, *Acinetobacter*, and *Achromobacter* sp. [53].

A total of 121 client-owned dogs were investigated for otitis media (OM) through MRI studies. In brief, the targeted MRI protocol effectively identified inflammation of the tympanic bulla mucosa and clearly distinguished avascular debris from vascularized soft tissue. The results revealed that OM was detected in 21% of the dogs with chronic otitis externa. The otoscopic findings showed an intact tympanic membrane in 15% of the samples, a rupture in 39%, and no visible membrane in 34%; however, the data was unavailable for 12% of the total samples. Cytology indicated that rods increased the likelihood of OM only when accompanied by inflammatory cells. Thus, across the studies, the trend suggested that occult OM can befall in cases of chronic OE, for which an MRI examination can be a useful diagnostic tool in their management [54].

In another study, 50 dogs with bilateral otitis externa were evaluated for a period of over 10 months. Standard microbiological techniques were used to inoculate the sterile swabs from both ears, and agar diffusion was used to assess the antibiotic susceptibility of *Staphylococcus intermedius* isolates. All the samples exhibited microbial proliferation; polymicrobial infections and the absence of anaerobes were also noted. Among the bacterial isolates, *Malassezia pachydermatis* and *S. intermedius* were the most commonly found. Significant variations in microbial isolates between the right and left ears were identified in 34 of the 50 dogs (68%, *p* < 0.05). Penicillin, ampicillin, erythromycin, tetracycline, and clindamycin were all highly resistant to *S. intermedius*. These data show that, in bilateral otitis externa, each ear should be cultured and examined independently to understand the nature of the infection precisely [55]. A similar study aimed to determine the prevalence of bacterial and yeast otitis externa in dogs and to assess the antimicrobial susceptibility. The dataset involved 257 ear swab samples collected from Bulgarian dogs. Bacteria were recovered in 93.77% of cases, which occurred as mono-infections, mainly as coagulase-positive staphylococci, *Malassezia pachydermatis* (109 cases) and *Pseudomonas aeruginosa*, and more commonly as mixed infections (132 cases). Rare isolates included β-haemolytic *Streptococcus* sp., *Proteus mirabilis*, and *Escherichia coli*, with the latter two detected nearly exclusively in mixed infections. Antimicrobial testing presented that Gram-positive bacteria were very susceptible to beta-lactams and aminoglycosides, while Gram-negative bacteria were responsive to aminoglycosides, polymyxin B, and enrofloxacin. Thus, the findings underscored the recurrent frequency of yeast and the necessity of performing antibiotic susceptibility testing together [56].

### 3.3. Mechanisms of Antimicrobial Resistance in Companion Animals

**Figure 3:**
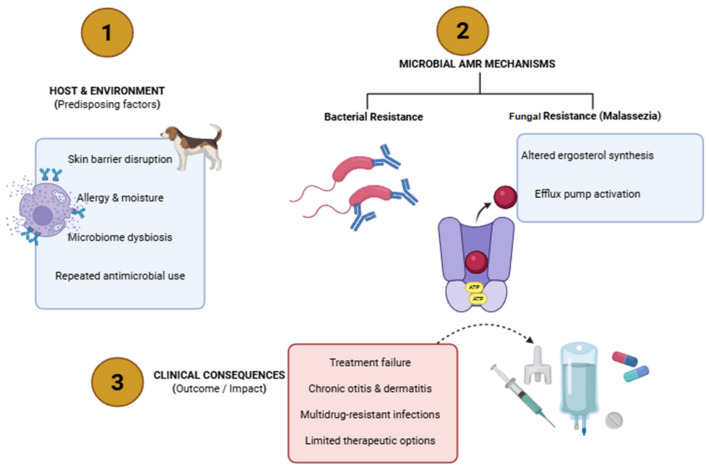
Illustration of the multifactorial mechanisms of antimicrobial resistance in the skin and ear infections of companion animals.

A significant concern is the recent increase in bacterial isolates that are *mecA*-positive and resistant to antibiotics. Additionally, many isolates exhibit multidrug resistance, which complicate the treatment and increase the risk of animal-to-human transmission [57]. Several MRSA and methicillin-resistant *S. epidermidis* lineages frequently reported in hospitals and communities have been identified, suggesting that companion animals may both host and disperse successful human-associated clones. Establishing clinical breakpoints and setting precise antibiotic usage recommendations are urgently needed [58]. Skin and ear infections in dogs and cats are commonly caused by a limited set of bacterial taxa—primarily *S. pseudintermedius* among Gram-positives, and *P. aeruginosa*, *E. coli*, and *Proteus* sp. among Gram-negatives [59]. These pathogens increasingly display MDR, complicating treatment and raising One Health concerns because of close contact between pets and people. Surveillance studies and hospital series continue to document the high rates of resistance and the frequent occurrence of MDR strains in clinical isolates from dermatologic and otic cases [60]. One study investigated the evolution of resistance to antimicrobials, along with the corresponding mechanisms and molecular characteristics of *Staphylococcus* sp. between 1999 and 2014. The study reported that 74 *staphylococcal* isolates (11.6%), including 11 MRSA (40.7%), 40 MRSP (8.7%), and 23 methicillin-resistant CoNS (26.7%), reported the presence of the *mecA* gene. The total number of isolates that tested positive for *mecA* increased significantly over time, and in parallel offered resistance to the majority of antibiotic classes. Three PFGE clusters, corresponding to ST22-IV, ST105-II, ST398-V, and ST5-VI, were generated by the MRSA isolates. The majority of methicillin-resistant *S. epidermidis* isolates were spread among eight PFGE clusters, which belonged to clonal complex 5, and included a recently identified sequencing type. Five PFGE clusters of the MRSP isolates were identified, including ST45-NT, ST71-II-III, ST195-III, ST196-V, ST339-NT, ST342-IV, and the recently identified ST400-III. Two PFGE clusters were ascribed to methicillin-resistant *S. haemolyticus* [61]. Figure 3 shows an illustration of the multifactorial mechanisms of antimicrobial resistance in the skin and ear infections of companion animals.

Animal skin and soft tissue infections (SSTIs) are frequently caused by *Staphylococcus aureus*, where another similar study reported AMR in *S. aureus* in specific cases. Antimicrobial and heavy-metal susceptibility, as well as the resistance genes, were investigated in 55 SSTI-associated isolates from companion animals collected during 1999–2018 (Lab 1) and 2017–2018 (Lab 2). Methicillin-resistant *S. aureus* (MRSA) accounted for more than half of the isolates (56.4%, 31/55), and multidrug resistance was present in 14.5%. The most frequent causes of resistance were β-lactams (81.8%), fluoroquinolones (56.4%), and macrolides/lincosamides (14.5%). Different non-wild-type subpopulations linked to particular resistance determinants were identified by heavy-metal MIC profiles. Although ST22-agrI predominated (45.5%, 25/55) and contained only MRSA, the isolates showed significant genetic heterogeneity. Other lineages also included ST5-agrII (14.6%), ST398-agrI (9.1%), and ST72-agrI (7.3%) [30]. According to a bacteriological analysis of canine otitis externa, the most frequent isolate was *Staphylococcus pseudintermedius* (41%), followed by *S. aureus* (23%) and *Pseudomonas aeruginosa* (19%); however, *Proteus mirabilis*, *Streptococcus canis*, and *Escherichia coli* were fewer common pathogens. *P. aeruginosa* was associated with a 90% recurrence rate, which reflected on the clinical challenged posed by Gram-negative opportunists, while the dominance of *S. pseudintermedius* highlighted its role in the infection. Moreover, the study reported that *P. aeruginosa* demonstrated significant resistance to β-lactams, exhibiting over 70% resistance to third-generation cephalosporins and 93% resistance to amoxicillin–clavulanate.

On the other hand, *P. mirabilis* showed reduced susceptibility to carbapenems, nitrofurantoin, and polymyxin, as well as complete resistance to tetracycline and moderate resistance to doxycycline. By contrast, *S. epidermidis* demonstrated extensive resistance to β-lactams, fluoroquinolones, sulfonamides, and polymyxins. Overall, the significant prevalence of multidrug-resistant bacteria in canine ear infections underscores the concerns about zoonotic dissemination and the transfer of antibiotic resistance genes, and further underscores the necessity of culture-based treatment approaches [62]. A recent study by Wang et al. [62] examined 896 samples of canine bacterial skin infections that were obtained for the period between 2018 and 2022 from the China Agricultural University Veterinary Teaching Hospital. Species identification was performed using 16S rRNA gene sequencing and MALDI-TOF. Amongst the bacterial populations, *Staphylococcus pseudintermedius*, *Pseudomonas aeruginosa*, and *Escherichia coli* were the most common bacteria isolated from 722 (80.6%) of the total samples. The broth microdilution method was adopted to study the antimicrobial susceptibility. AMR was notable in *E. coli* against ceftriaxone from 30.0% to 72.7% and florfenicol in *S. pseudintermedius* from 9.1% in 2018 to 20.0% in 2022. The most predominant infection type among the 305 cases that were evaluated was pyoderma (47.5%, 145/305), which was mostly linked to *S. pseudintermedius*. Otitis (25.6%), which was primarily linked to *P. aeruginosa* and *S. pseudintermedius*, was the most often isolated species in both single and mixed infections (35.4%). Interestingly, eight dogs were infected with identical strains of *S. pseudintermedius*, reported by a core-genome SNP analysis [63]. Earlier studies have also reported for AMR in cats. A similar study reported the susceptibility of major pathogens from antimicrobial non-treated animals with critical clinical symptoms of skin, wound, or ear infections for the period 2008–2010. The results reported that *Staphylococcus pseudintermedius*, *Pasteurella multocida*, and *Staphylococcus pseudintermedius* were most common. The resistance capacity to penicillin, clindamycin, and chloramphenicol varied from 18.4% to 25.2% for *Staphylococcus pseudintermedius*; however, the resistance was lower for ampicillin, amoxicillin, clavulanate, and fluoroquinolones. Resistance to beta-lactam was low but higher, up to 62.1%, for *Staphylococcus aureus.* MecA-positive results were obtained for 6.3% of *Staphylococcus pseudintermedius* and for 5.4% of *Staphylococcus aureus* cases. *Pseudomonas aeruginosa* was fairly disposed to the effects of gentamicin and fluoroquinolones. Penicillin, ampicillin, chloramphenicol, and fluoroquinolone resistance was either non-existent or extremely low for *Streptococci*, while *E. coli* showed little resistance to gentamicin, chloramphenicol, and fluoroquinolones [64].

Bacterial and fungal infections are complex and multifaceted in the etiology of otitis in dogs and cats. Hamdy et al. [64] characterized the microbiological characteristics and susceptibility profiles of bacterial pathogens linked to 212 cases of external otitis, which included 94 cats and 118 dogs. Standard microbiological techniques were adopted to investigate the ear samples, followed by antibiotic sensitivity profiling and PCR screening for enlisting virulence and resistance genes. The isolates were resistant to amoxicillin (3.8%), amoxicillin/clavulanic acid (55.4%), ceftriaxone (26.6%), ceftazidime (27.5%), cefoperazone (22.9%), enrofloxacin (1.6%), gentamicin (9.9%), amikacin (7.2%), linezolid (92.8%), and tylosin (76%). ESBL genes, such as the *bla*_TEM_ and *bla*_CTX-M_ gene, along with other resistance genes, like the *aadB* and *qnrB* gene, were identified in *E. coli* and *K. pneumoniae* according to PCR screening of the isolated bacteria. The same resistance genes were also present in the other Gram-negative isolates of *P. aeruginosa* and *P. mirabilis*. The *blaZ* and *norA* resistance genes were present in gram-positive *S. aureus*. Thus, the study suggested that customized monitoring algorithms aid in identifying specific AMR frameworks, provide upkeep for physicians in making rational treatment decisions, and limit the selection and spread of AMR within the population [65]. The insights of the above findings suggest that, from a One Health perspective, the clinical significance extends beyond individual cases. Resistance determinants are frequently disseminated via horizontal gene transfer, enabling the spread of resistance across bacterial species and between animals, humans, and the environment. Close human–pet interactions and shared environments further facilitate this exchange, positioning companion animals as important reservoirs and amplifiers of resistant organisms. Consequently, understanding the resistance mechanisms is essential not only for guiding targeted therapy but for informing integrated surveillance, antimicrobial stewardship, and infection control strategies across the One Health continuum. Table 3 depicts the mechanisms of antimicrobial resistance in skin and ear infections for dogs and cats. As a critical note, it is significant to convey that AMR in companion animal pathogens is driven by key mechanisms such as enzymatic inactivation, target modification (e.g., *mecA*-mediated resistance), reduced permeability, and active efflux, often co-occurring to produce MDR. The rapid spread of these traits is facilitated by horizontal gene transfer, while biofilm formation further enhances tolerance and persistence, particularly in chronic infections. Clinically, these mechanisms result in treatment failure, recurrence, and reduced efficacy of empirical therapy, increasing the reliance on last-line antimicrobials. From a One Health perspective, their mobility and persistence enable cross-species dissemination across animal, human, and environmental interfaces. Together, these factors underscore the need for susceptibility-guided therapy, targeted interventions, and strengthened antimicrobial stewardship.

### 3.4. Therapeutic Implications of AMR

The effectiveness and clinical viability of innovative treatments, such as bacteriophage therapy, antimicrobial peptides (AMPs), nanoparticle-based systems, and CRISPR-Cas technologies, against MDR bacterial infections in cats and dogs are assessed using a mixed-methods strategy. High resistance rates were found in retrospective clinical data from 512 cases, particularly in *Pseudomonas aeruginosa* and *Staphylococcus pseudintermedius*, with multidrug resistance in certain isolates above 80%. Antibiotics with minimal inhibitory values of 0.05–0.10 µg/mL effectively cured 85% of the 120 MDR isolates examined in vitro. Silver nanoparticles and chitin-based nanofiber composites exhibited strong antibacterial activity despite their little cytotoxicity. Compared to standard antibiotics phage–antibiotic treatment produced the highest cure rate, with good swiftness up to 82% and an average healing period of 10 days. Phage treatment and AMPs appeared to be safe and effective, according to the majority of the forty veterinary groups, while CRISpen-Cas and nanotechnology raised the safety concerns. In order to address the increase in antibiotic resistance, all of these findings suggest that veterinary dermatology should incorporate alternative medications. These adoptive strategies provided a systematic approach to deploy cutting-edge techniques for managing pet diseases, particularly those that veterinarians approve of [65]. Table 4 presents the therapeutic implications of AMR in skin and ear infections in companion animals.

#### 3.4.1. Role of Antimicrobial Peptides (AMPs)

AMPs are cationic, amphipathic peptides and innate immune effectors synthesized in almost all living organisms; although there are many varieties, defensins and cathelicidins are two important groups in mammals that are expressed by leukocytes and epithelial cells [78]. AMPs play a variety of roles, including enticing additional immune cells and targeting direct impacts on germs and fungus. Since defensins have the potency to directly bind to the cell membrane of pathogens, and moreover since they are positively charged, they are drawn into the negatively charged cell membrane and are paired up to form a pore, which leads to cellular breakdown and death. Up to 10% of people and dogs suffer from atopic dermatitis (AD), a chronic inflammatory skin condition brought on by an allergic reaction to nonpathogenic environmental antigens. Researchers are able to draw relevant comparisons because of the clinical and histological similarities between AD in humans and dogs [79]. The existence of a malfunctioning epidermal barrier is a commonality between AD patients in humans and dogs. Secondary bacterial infections, such as *Staphylococcus pseudintermedius* in dogs and *S. aureus* in humans, can take advantage of the compromised barrier to colonize and exacerbate skin lesions in AD patients [80]. AMPs, called β-defensins and cathelicidins (LL-37), are found in the human skin’s innate immune system. These peptides are insignificant in healthy skin, but they build up in skin afflicted by inflammatory conditions like psoriasis [81].

**Figure 4:**
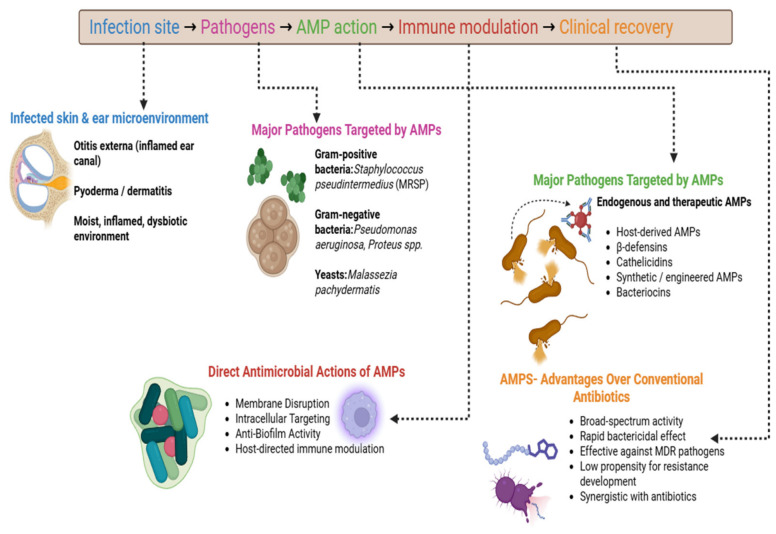
Role of antimicrobial peptides in the management of otitis externa and skin infections in dogs and cats.

The trends across the studies have suggested that there are high expression levels of human β-defensin 2 (HBD-2) and LL-37 in the inflammatory skin from psoriasis and atopic dermatitis patients. All psoriasis subjects exhibited high levels of LL-37 and HBD-2 in their superficial epidermis, as per immunohistochemical investigation. By contrast, subjects with AD had significantly lower immunostaining for these peptides in both acute and chronic lesions. Moreover, Western blot and immunodot blot analyses reinforced these findings. RT-PCR examinations revealed that HBD-2 mRNA and LL-37 mRNA were substantially less expressed in atopic lesions than in psoriatic lesions. The elimination of *S. aureus* was possible with the combination of LL-37 and HBD-2, which demonstrated synergistic antibacterial action. The microbial diversity of AD skin is lower than that of healthy skin. This is due to the reason that members of the commensal *Staphylococci* exhibited anti-*S. aureus* activity, which impacted on the decreased abundance of the genera *Streptococcus, Corynebacterium*, and *Cutibacterium* [82]. The response of the immune system to infections is based on the bacterial populations. It has been reported that the genus *Corynebacterium* stimulated IL-23 signaling and produced IL-17A γδ T cells in the dermis, which involved more immune cells. It has been demonstrated that *Cutibacterium acnes*, which is linked to healthy skin, triggered the release of extracellular traps from certain Th17 subsets at the species level [83]. The negative coagulase activity of *Staphylococcus epidermidis* in mouse skin was reported to improve innate protection against *Candida albicans* by upregulating Th17 immune mediators like S100A8 and S100A9 [84], whereas *Staphylococcus cohnii* activated the host steroid pathway and promoted immunosuppression [85]. On the other hand, commensal microorganisms vital for the formation of the epidermal barrier regulated the surface pH [86]. For instance, studies have reported that *Staphylococcus epidermidis* increased the production of skin ceramides, whereas *Roseomonas mucosa* produced glycerophospholipids that induced host epithelial repair by enhancing cholinergic activation via TNFR2 signaling, and *Cutibacterium acnes* secreted a specific lipase that converted triacylglycerols in sebum to propionic acid, which contributed to the acidification of the skin surface to inhibit the growth of *S. aureus* [87]. Figure 4 shows a schematic illustration of the role of AMPs in the treatment of otitis externa and skin infections in dogs and cats. AMPs derived from host or synthetic sources exert rapid antimicrobial activity through membrane disruption, intracellular targeting, and biofilm inhibition, while simultaneously modulating host immune responses and promoting tissue repair. These multifaceted actions make AMPs promising alternatives or adjuncts to conventional antibiotics, particularly against multidrug-resistant pathogens.

#### 3.4.2. Role of Bacteriophage Therapy

The rising application of antimicrobials in pets, especially dogs, involving broad-spectrum agents utilized in human healthcare, fosters antibacterial resistance, particularly in MDR bacteria like *Pseudomonas aeruginosa*, *Staphylococci*, and Enterobacteriaceae. However, this concern must no longer be overlooked in veterinary medicine for dogs [88]. The successful treatment of chronic *P. aeruginosa*-associated otitis externa in dogs was one of the first known uses of phage therapy in companion animals [89]. Topical treatment of a phage formulation indicated therapeutic advantages and pathogen clearance in sites where standard antibiotic regimens failed because of resistance [90]. The data demonstrated the viability of phage therapy for canine infections that are resistant to treatment [89]. In a similar study [91], ten dogs with persistent *Pseudomonas aeruginosa* otitis were enrolled in an assessment of a bacteriophage therapy. A single dosage of a topical preparation containing roughly 1 × 10^5^ plaque forming units (PFU) of each of six bacteriophage strains that are active against *P. aeruginosa* was administered directly into the auditory canal of one ear and the temperature was monitored. The results reported that the clinical score from the aural swabs and *P. aeruginosa* count of every ear decreased, with the increase of the bacteriophage count during 48 h post treatment without any side effects like inflammation. Therefore, these findings demonstrated the impact of a topical bacteriophage mixture against *P. aeruginosa* infections without showing any signs of toxicity, and can perhaps be a practical and successful treatment for *P. aeruginosa* otitis in dogs [91]. In a recent study, Antoine et al. [92] used a *Galleria mellonella* larvae model to investigate the efficiency of the PEV2 phage against a clinical *P. aeruginosa* isolate from a canine otitis. The PAV237 *P. aeruginosa* isolate’s genomic DNA was decoded and documented. The larvae survival rate over a 4-day period was used to evaluate the effectiveness of the PEV2 phage against PAV237 at various stages of infection. In the experimental groups of the infected larvae, PEV2 therapy demonstrated high boost and survival rates. Kaplan–Meier curves demonstrated that the rate of alive larvae was noticeably greater in the non-infected larvae than in the infected-treated larvae. Moreover, phage titers increased with the multiplication of infection, however *P. aeruginosa* titers decreased at 24 and 48 h. Thus the in vivo study revealed that PEV2 was potent and active against *P. aeruginosa*, and additionally PEV2 replication was related with a decreased bacterial proliferation in the phage-treated larvae. Bacteriophages with potent in vitro lytic activity against a variety of pathogenic *P. aeruginosa* strains were identified and characterized by Santos et al. [93]. Dogs with spontaneous ulcerative keratitis rich in *P. aeruginosa* strains were infected with two bacteriophages (P2S2 and P5U5) and determined the host ranges, phage nucleic acid type, and genetic profile. In addition, electron microscopy, temperature, co-cultivation, and pH stabilities were also studied to report the host bacterial growth. The titers of both bacteriophages dropped after heating for 10 to 50 min at 45 or 60 °C, but the bacteriophages endured stability in the pH range of 4 to 12. In the co-culture with P2S2 or P5U5, the growth of each *P. aeruginosa* isolate was markedly suppressed, which indicated the dose response correlation with the plaque-forming unit-to-CFU ratios [93]. Phage therapy has drawn a lot of interest as an alternative to antibiotic treatment with the rise of antibiotic-resistant bacteria, including methicillin-resistant *Staphylococcus aureus* (MRSA) and methicillin-resistant *Staphylococcus pseudintermedius* (MRSP) [94]. Table 5 shows the pathogen-wise bacteriophage therapy for AMR skin and ear infections (dogs and cats).

Kayvirus bacteriophages are severely lytic in *Staphylococcus* species and have a broad host range. Kayvirus ɸSA039 exhibited a specific host-recognition mechanism which is unique from that of other known kayviruses [98]. Azam et al. [99] reported that SA039 acquired a mutation in open reading frame (ORF) 100 and ORF102, which led to swap the receptor and infect *S. aureus* without the β-GlcNAc residue. In general, most kayviruses use the backbone of wall teichoic acid (WTA) as their receptor for binding. *S. pseudintermedius* differed from *S. aureus* due to modification of the WTA structure, which may lead to infection by SA039. By contrast, the results implied that glycosylation in the WTA of *S. pseudintermedius* was crucial for the adsorption of *S. pseudintermedius*-specific phages, but not for SA039. Particularly, glycosylation of ribitol phosphate (RboP) WTA by TarM or/and TarS inhibited the infection of *S. aureus* by SP phages. Thus, the study demonstrated a novel strategy for the treatment of *S. aureus* and *S. pseudintermedius* infection and provided valuable insights into the biology of phage–host interactions [100]. By evaluating the in vitro lytic capacity of 40 naturally occurring bacteriophages on 53 uropathogenic *E. coli* (UPEC), Junjappa et al. [100] examined the viability of using bacteriophage therapy to treat canine and feline *E. coli* urinary tract infections (UTIs). The study reported that a single bacteriophage lysed an average of 40% of the UPEC strains. Overall, one or more of the bacteriophages killed 94% of the UPEC strains. About 92% of the UPEC strains collectively and 51% of the UPEC strains individually were lysed by ten bacteriophages. A majority of the bacteriophages were lytic T4-like genus, while fewer exhibited morphologic resemblances to temperate P2-like bacteriophages. Overall, these findings suggested that most UPEC was lysed by naturally occurring bacteriophages, which could be taken as potential therapeutic agents for the management of *E. coli*. Similar studies have been reported for cats. Braunstein et al. [95] investigated the multidrug-resistant *P. aeruginosa* implant-associated in the post-operative illness of arthrodesis in Siamese cats with a customized phage–antibiotic treatment. Ceftazidime and anti-*P. aeruginosa* phage specifically harmonized to the pathogen comprised the treatment. This tailored phage therapy in cats demonstrated that the efficacy of personalized phage–antibiotic therapies against persistent infections in veterinary practice are made possible by the effective outcome.

#### 3.4.3. Role of Nanoparticle-Based Systems

**Figure 5:**
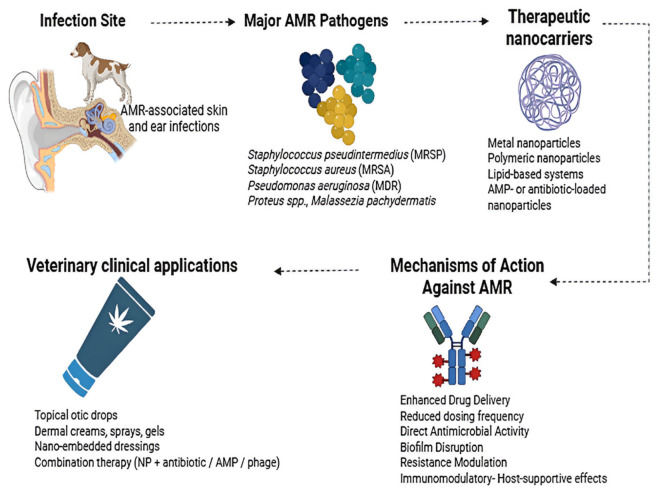
Role of nanoparticle-based systems in combating antimicrobial resistance in skin and ear infections of companion animals.

In contrast to antibiotics, which frequently target certain bacterial enzymes or structures, nanoparticles (NPs) function through strategies that are essentially different. Metallic nanoparticles have the capacity to physically damage cell membranes and produce reactive oxygen species (ROS) that impair cellular constituents which eventually obstruct the growth of microorganisms [101]. Compared to single-target antibiotics, this complex approach lessens the possibility that bacteria would rapidly develop resistance. With advantages such as prolonged systemic circulation, upgraded bioavailability, reduced toxicity and enhanced therapeutic efficiency, the potential of NPs as a drug delivery system in veterinary medicine has been extensively studied [102]. Figure 5 shows a schematic representation of nanoparticle-based therapeutic systems applied to combat antimicrobial resistance in skin and ear infections of companion animals. Nanocarriers enhance drug delivery, disrupt biofilms, exert direct antimicrobial effects, and modulate host responses, thereby improving treatment efficacy against multidrug-resistant pathogens and reducing reliance on conventional antibiotics.

The commensal bacteria *Staphylococcus pseudintermedius* is commonly isolated from dog skin and is known to be a zoonotic agent, particularly for dog owners. Seo et al. [103] investigated the antibiofilm activity of silver nanoparticles (AgNPs) against the *S. pseudintermedius* obtained from dogs with otitis externa. The experimental results revealed that 20 and 10 µg/mL concentrations of AgNPs demonstrated substantial dose-dependent antibiofilm efficacy and decreased biofilm formation. Particularly at 20 µg/mL, AgNPs produced less bacterial slime with morphological cellular disruptions indicating cell lytic activity and suggesting that AgNPs may be an effective antibiofilm agent for canine otitis externa. In a similar study, Meroni et al. [104] investigated the *S. pseudintermedius* phenotypes of antibiotic resistance, the capacity of its biofilm formation, and the spread of virulence factors. Standard techniques, such as the Kirby–Bauer assay, were used for the examination and identification of 73 *S. pseudintermedius* strains for antibiotic resistance against 22 distinct compounds. The microtiter plate assay and the amplification of the *icaA* and *icaD* genes were opted to unveil the biofilm formation. The results portrayed that antibiotic-resistance and multi-drug resistant profiles were identified for 43% and 57% of the strains, respectively. Biofilm producers were produced by all MDR strains and 8/31 (27%) non-MDR strains. Of the isolates, leukotoxin LukI, enterotoxin gene *seC*, *expA*, and *expB* were detected by 96%, 64%, 4%, and 7%, respectively. Hence, the study reported that *S. pseudintermedius* as a potential biofilm forming human and zoonotic agent with the potential of horizontal gene transfer with virulence characteristics.

## 4. Future Challenges and Perspectives

Firstly, the increasing incidence of MDR infections in companion animals is a main issue. High rates of resistance have been found in common clinical isolates, such as *E. coli* [105], *Staphylococcus pseudintermedius*, and other Gram-negative bacteria. These organisms recurrently show resistance to the vital antimicrobials used in veterinary and human medicine, making treatment more challenging and increasing morbidity and treatment failure [76]. Since resistance is dynamic, it is challenging to forecast future trajectories. Accurately tracking the dissemination of pertinent resistance genes across topographical regions will continue to be difficult in the absence of reliable monitoring and reporting methods. This makes it more difficult to act quickly and could lead to the silent spread of resistant clones. Secondly, companion animals are frequently less concentrated in terms of systematic surveillance, in contrast to livestock, for which many nations have set up national AMR surveillance networks.

The data are dispersed, unreliable, and mostly sourced from specific hospitals or research laboratory reports rather than integrated national databases due to the absence of regular, required monitoring standards [106]. As a result, there are substantial gaps in our understanding of the actual prevalence of AMR in pet populations [107]. Therefore, timely identification, diagnosis, and containment of developing resistant strains will be dependent on the establishment of standardized national and international surveillance techniques that integrate pets and are in line with One Health frameworks [76]. Thirdly, due to financial limitations, a lack of quick diagnostics, and time constraints, antibiotics are occasionally provided empirically without culture and susceptibility testing. Additionally, even in cases when there is little clinical indication of a bacterial infection, pet owners frequently expect antibiotics, which may encourage needless use [108]. The interdependence of environmental, animal, and human health heightens the inevitability of coordinated strategies. The progression of animal and human antimicrobial stewardship initiatives may be hampered by inadequate segregation. Resistant strains that are prevalent in homes/shelters can reinfect communities or animals and increase the burden of resistance at the community level [109]. A promising future path is the combination of CRISPR-Cas technology with conventional veterinarian antibiotic stewardship techniques [110]. Few applications include the therapeutic CRISPR antimicrobial medicines designed to target particular MDR infections in companion animals, as well as adopting diagnostic instruments that rapidly identify resistance genes in clinical samples by utilizing CRISPR’s specificity [110]. Further, microbiome editing reduces harmful MDR strains by selectively varying bacterial communities in the skin or gut using CRISPR technologies [29,111].

To put in a nutshell, key barriers continue to limit the effective control of AMR in companion animal dermatology and otology. Limited access to culture and susceptibility testing—due to cost and turnaround time—drives empirical therapy and suboptimal outcomes. The rising prevalence of multidrug-resistant pathogens, including methicillin-resistant *S. pseudintermedius* and resistant Gram-negative bacteria, is rapidly narrowing therapeutic options. Persistent overuse of systemic antimicrobials, alongside an underutilization of topical therapies, further accelerates resistance selection. In parallel, fragmented One Health surveillance restricts the tracking of AMR across animal, human, and environmental interfaces. The management of chronic and recurrent infections remains challenging due to underlying conditions, biofilm-associated persistence, and compliance issues. Addressing these gaps requires improved diagnostic accessibility, targeted stewardship, greater adoption of non-systemic therapies, and integrated One Health surveillance strategies.

## 5. Conclusions

Controlling the emergence and spread of antimicrobial resistance (AMR) would require harmonizing antimicrobial use laws and regulations worldwide with precise guidance for companion animal operations. The dangers of AMR, appropriate antibiotic use, and the implication of preventative care, including immunization and hygiene practices, should be highlighted in educational programs intended at pet owners, veterinary students, and practicing veterinarians. Further, clinical practice, behavioral drives, surveillance, research, legislation, and international public health are just a few of the many issues surrounding antimicrobial resistance in companion animals. Integrated One Health methods, such as thorough surveillance, upgraded stewardship, cutting-edge treatments, and cooperative policy formulation, are essential for the future. Upholding antibiotic efficacy and defending the health of people, pets, and ecosystems will require funding for research, education, and coordinated action. On a concluding note, clinicians should prioritize susceptibility-guided therapy, adopt topical-first approaches where appropriate, limit systemic antimicrobial use, and address underlying conditions to prevent recurrence. Vigilance for multidrug-resistant pathogens, adherence to evidence-based treatment protocols, and integration of infection control and One Health principles are essential to optimize outcomes and to mitigate antimicrobial resistance.

## Figures and Tables

**Table 1:** Common Infections in Companion Animals—Microbial Etiology and AMR Profiles.

Disease Focused	Major Microbial Agents	Antimicrobial Resistance (AMR) Profiles Reported	Reference
Canine pyoderma & otitis externa	*Staphylococcus pseudintermedius*, *S. aureus*, *Pseudomonas aeruginosa*, *Enterococcus* sp.	High resistance to β-lactams (penicillin, amoxicillin); increasing methicillin-resistant *S. pseudintermedius* (MRSP); fluoroquinolone and aminoglycoside resistance in *P. aeruginosa*.	[9]
Dermatophytosis	*Microsporum canis*, *Trichophyton* sp.	Antimicrobial resistance not applicable; reports emerging reduced susceptibility to azole antifungals in chronic cases.	[29]
Canine otitis externa	*S. pseudintermedius*, *S. aureus*, *P. aeruginosa*	MRSP and MRSA prevalent; resistance to enrofloxacin, gentamicin, and cephalosporins; *P. aeruginosa* often MDR.	[30]
Zoonotic companion animal-associated AMR infections	*Escherichia coli*, *Klebsiella pneumoniae*, *Salmonella* spp.	ESBL production, carbapenem resistance, fluoroquinolone resistance, methicillin resistance; evidence of transmission between pets and humans.	[31]
Multicenter companion animal UTIs	*Escherichia coli*, *Proteus mirabilis*, *Klebsiella* spp.	Widespread resistance to ampicillin and sulfonamides; increasing resistance to fluoroquinolones and third-generation cephalosporins; MDR Enterobacteriaceae.	[32]
Companion animal urinary tract infections (UTIs)	*Escherichia coli*, *Enterococcus* spp., *Klebsiella pneumoniae*, *Staphylococcus pseudintermedius*	High resistance to ampicillin, amoxicillin–clavulanate, fluoroquinolones, and trimethoprim–sulfamethoxazole; emergence of ESBL-producing Enterobacteriaceae and MDR urinary isolates.	[33]
Surgical-site and wound infections in dogs and cats	*S. pseudintermedius*	Resistance against critically important human antimicrobials including cephalosporins and fluoroquinolones; zoonotic MDR strains reported.	[34]
Respiratory tract infections in dogs and cats	*Pasteurella multocida*, *Bordetella bronchiseptica*, *Staphylococcus aureus*	Reduced susceptibility to tetracyclines and β-lactams; emerging MDR respiratory isolates complicate empirical treatment.	[35]

**Table 2:** Bacterial Pathogens Associated with Skin Infections in Dogs and Cats.

Bacterial Pathogen	Animal Type	Common Skin Conditions	Key Clinical Notes	Major Antimicrobial Resistance (AMR) Concerns	References
*Staphylococcus pseudintermedius*	Dogs (primarily), Cats (occasional)	Superficial and deep pyoderma, wound infections	Most common cause of canine bacterial skin infections	Methicillin-resistant *S. pseudintermedius* (MRSP); resistance to β-lactams, fluoroquinolones, macrolides.	[44]
*Staphylococcus aureus*	Dogs and Cats	Pyoderma, abscesses	Zoonotic potential; less common than *S. pseudintermedius*	MRSA; multidrug resistance.	[30,45]
*Staphylococcus schleiferi*	Dogs	Otitis externa, pyoderma	Often misidentified; clinically significant	Methicillin resistance reported.	[46]
*Pseudomonas aeruginosa*	Dogs and Cats	Deep pyoderma, chronic wounds	Frequently associated with chronic or recurrent infections	Intrinsic MDR; resistance to fluoroquinolones and aminoglycosides.	[44,47]
*Escherichia coli*	Dogs and Cats	Wound and post-operative infections	Opportunistic pathogen	ESBL production; multidrug resistance.	[48]
*Klebsiella pneumoniae*	Dogs and Cats	Abscesses, wound infections	Opportunistic and nosocomial	ESBL-producing and MDR strains.	[49]
*Streptococcus* sp.	Dogs and Cats	Cellulitis, necrotizing infections	Can cause acute, severe disease	Resistance to macrolides and tetracyclines.	[48]

**Table 3:** Mechanisms of Antimicrobial Resistance in Skin and Ear Infections (Dogs and Cats).

AMR Mechanism	Molecular/Phenotypic Basis	Associated Pathogens	Implications in Companion Animal Infections	References
β-lactamase & ESBL production	Production of β-lactamases and extended-spectrum β-lactamases (e.g., CTX-M, TEM)	*Escherichia coli*, *Klebsiella pneumoniae*	Hydrolyze penicillins & cephalosporins; limits efficacy of β-lactams in skin and wound infections.	[48]
Methicillin resistance (PBP alteration)	*mecA* or *mecC* gene altering penicillin-binding proteins	*Staphylococcus pseudintermedius*, *Staphylococcus aureus*	Confers resistance to methicillin/oxacillin and most β-lactams; difficult to treat pyoderma/otitis.	[66]
Efflux pumps	Overexpression of efflux systems reducing intracellular drug levels	*Pseudomonas aeruginosa*, *Staphylococcus* sp.	Contributes to multidrug resistance in chronic infections.	[67]
Biofilm formation	Biofilm matrix protecting bacteria from antibiotics and host defenses	*Staphylococcus pseudintermedius*, *Pseudomonas aeruginosa*	Biofilms increase chronicity and recurrence of skin and ear infections.	[67,68]
Target site modifications (gyrA/parC)	Mutations in DNA gyrase/topoisomerase	*Pseudomonas* sp., fluoroquinolone-resistant staphylococci	Reduced susceptibility to fluoroquinolones.	[69]
Virulence-associated toxin production	Presence of leukocidin genes (*lukS*, *lukF*) and exfoliative toxin gene (*siet*) along with biofilm genes	*Staphylococcus pseudintermedius* ST2660	Enhances tissue damage, immune evasion, and severity of dermatological and abscess-forming infections in companion animals.	[67]
Multidrug resistance (MDR) phenotypes	Combination of multiple mechanisms conferring resistance to ≥3 drug classes	*Staphylococcus pseudintermedius*, *Pseudomonas aeruginosa*, *E. coli*	Limits therapeutic options; necessitates culture-guided therapy.	[70]
Intrinsic resistance	Chromosomal determinants and low permeability	*Pseudomonas aeruginosa*	Intrinsic resistance to many antibiotics, notably β-lactams and fluoroquinolones.	[68]

**Table 4:** Therapeutic Implications of AMR in the Skin and Ear Infections of Dogs and Cats.

Therapeutic Implication	Description	Clinical Impact in Dogs & Cats	Reference
Need for antimicrobial stewardship (AMS)	Judicious use of antibiotics guided by diagnostics rather than empirical empiricism	Improves treatment success and slows resistance emergence; reduces inappropriate use.	[71]
Culture & susceptibility testing before therapy	Use of laboratory diagnostics to tailor antimicrobial therapy	Reduces therapeutic failure, avoids use of ineffective drugs.	[72]
Topical therapy as first-line in superficial infections	Topical antiseptics/antibiotics can suffice for surface pyoderma	Limits systemic antibiotic exposure and reduces AMR selection.	[73]
Updated empirical therapy recommendations	Revised dosing & drug selections based on current resistance trends	Improves initial therapeutic outcomes while awaiting AST results.	[74]
Alternative & adjunctive therapies	Use of natural compounds, AMPs, phytochemicals, host-directed therapies	Potential to reduce reliance on conventional antibiotics and bypass common resistance mechanisms.	[75]
One Health and interdisciplinary approaches	Integrating veterinary, human, environmental insights for therapy choices	Reduces zoonotic transfer of resistant pathogens; supports broad stewardship.	[76]
Local resistance surveillance to inform therapy	Continuous collection of AMR data in practice	Allows evidence-based empirical therapy tailored to regional patterns.	[47]
Owner compliance & education	Ensuring proper dosing/duration and follow-up	Essential to avoid subtherapeutic exposure that selects resistant strains.	[77]

**Table 5:** Pathogen-wise Bacteriophage Therapy for AMR Skin and Ear Infections (Dogs and Cats).

Target Pathogen	Host/Infection Type	Study Design	Phage Strategy	Key Outcomes (AMR Relevance)	Reference
*Pseudomonas aeruginosa* (MDR)	Cat implant-associated skin infection	Clinical case report	Personalized phage + antibiotic therapy	Complete resolution of infection refractory to antibiotics; demonstrates safety and feasibility in cats.	[95]
*Pseudomonas aeruginosa* (MDR)	Dog-chronic otitis externa	In vitro + formulation + translational study	Phage cocktail with stabilizing excipients	Significant reduction in MDR *P. aeruginosa* load; effective biofilm disruption; improved therapeutic stability for otic use.	[96]
*Staphylococcus pseudintermedius* (MRSP)	Dog—superficial pyoderma	Clinical case report	Topical phage therapy	Complete clinical cure of MRSP pyoderma; reduced reliance on systemic antibiotics.	[97]
*Staphylococcus* sp. (MDR)	Dogs & cats skin and ear infections	Review (veterinary focus)	Phage monotherapy & phage–antibiotic synergy	Highlights strong anti-staphylococcal phage efficacy; reduced resistance emergence compared to antibiotics.	[43]
MDR bacteria (*Pseudomonas*, *Staphylococcus*)	Dogs & cats skin infections	Retrospective clinical analysis	Phage–antibiotic combinations	Higher cure rates vs. antibiotics alone in MDR infections; supports adjunct phage use.	[65]

## Data Availability

No new data were created or analyzed in this study. Data sharing is not applicable.
